# Exploiting the T790M Gatekeeper: A Theoretical Blueprint for Non-Covalent Inhibition of in *cis* Triple-Mutant EGFR

**DOI:** 10.3390/pharmaceutics18070842

**Published:** 2026-07-10

**Authors:** Shrikant S. Nilewar, Shuvadip Khanra, Manav Pandya, Sandesh Lodha, Perli Kranti Kumar, Nagaraju Bandaru, Antonio Jose Naranjo-Redondo, Ricardo Pérez-Pastén-Borja, Tushar Janardan Pawar

**Affiliations:** 1 Department of Pharmaceutical Chemistry, Maliba Pharmacy College, Uka Tarsadia University, Bardoli 394350, Gujarat, India; shrinilewar@gmail.com (S.S.N.); shuvadipkhanra@gmail.com (S.K.); pandyamanav49@gmail.com (M.P.); sandeshlodha@gmail.com (S.L.); 2 Department of Pharmaceutical Analysis, J.K.K. Nattraja College of Pharmacy, Kumarapalayam 638183, Tamil Nadu, India; drpkk1987@gmail.com; 3 Department of Pharmacology, Sree Dattha Institute of Pharmacy Sheriguda, Ibrahimpatnam, Hyderabad 501510, Telangana, India; bnrajupharma@gmail.com; 4 División de Ingeniería, Universidad Anáhuac Querétaro, Circuito Universidades I, Fracción 2 S/N, Zibatá, El Marqués 76246, Querétaro, Mexico; antonio.naranjo@anahuac.mx; 5 Departamento de Farmacia, Escuela Nacional de Ciencias Biológicas, Instituto Politécnico Nacional, Unidad Académica Adolfo López Mateos, Del. GAM, Mexico City 77380, Mexico; 6 Centro de Investigación, Universidad Anáhuac Querétaro, Circuito Universidades I, Fracción 2 S/N, Zibatá, El Marqués 76246, Querétaro, Mexico

**Keywords:** EGFR T790M, molecular dynamics, generative artificial intelligence, sulfur anchoring, drug resistance, free energy landscape, non-covalent inhibitors

## Abstract

**Background/Objectives:** The EGFR T790M mutation drives lung cancer resistance by sterically hindering inhibitors and restoring ATP affinity. As C797S mutations render covalent inhibitors obsolete, novel non-covalent strategies are critical. This study identifies inhibitors that redefine the mutant methionine sulfur as a primary stabilizing anchor rather than a liability. **Methods:** A generative AI framework (DrugEx) sampled 100,000 molecules, prioritized via QSAR classification (ROC-AUC: 0.91 ± 0.01) and Applicability Domain (AD) mapping. The workflow was de-risked through retrospective benchmarking against the DUD-E database (35,590 molecules), achieving a 1% Enrichment Factor of 5.19. Lead candidates underwent 100 ns all-atom molecular dynamics (MD) simulations. Mechanistic stability was quantified via Free Energy Landscape (FEL) analysis and ensemble-averaged MM-GBSA binding free energy calculations. **Results:** Candidate **106** demonstrated exceptional mutation tolerance by redistributing interactions toward the Met790 sulfur atom. MD analysis confirmed potency is dictated by successful recruitment of the thioether environment, locking the complex within a narrow thermodynamic basin. Candidate 106 maintained stable binding (−11.0 kcal/mol) corroborated by an equipotent MM-GBSA ΔG_bind_ of −50.51 kcal/mol in the mutant system, driven by persistent π-sulfur contacts (85% occupancy). **Conclusions:** These results indicates that potential T790M resistance bypass is achievable by exploiting the gatekeeper methionine’s electronic environment. This modeled mutation-aware framework provides a candidate non-covalent strategy to be validated in future wet-lab campaigns.

## 1. Introduction

The clinical management of non-small cell lung cancer (NSCLC) has been fundamentally redefined by the discovery of oncogenic drivers within the kinase domain of the epidermal growth factor receptor (EGFR) [1,2]. The advent of tyrosine kinase inhibitors (TKIs) targeting these sensitizing mutations, such as exon 19 deletions and the L858R point mutation, initially provided a paradigm shift in patient survival, offering robust and selective therapeutic responses [3,4]. These small molecules function by competing with adenosine triphosphate (ATP) for binding within the catalytic cleft, thereby preventing the phosphorylation of downstream signaling proteins that drive uncontrolled cellular proliferation [5]. However, the clinical utility of these agents is perennially truncated by the inevitable emergence of acquired resistance, which remains the most formidable obstacle in precision oncology [6,7].

**Figure 1:**
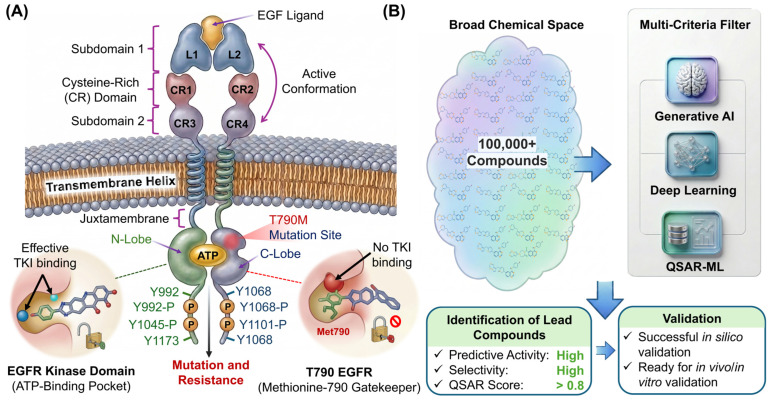
Translational landscape and mechanistic basis for mutation-aware EGFR inhibition. (**A**) Represents the progression of clinical resistance in NSCLC from sensitizing mutations to the T790M gatekeeper variant. (**B**) Details the discovery workflow for lead compounds.

Among the various resistance pathways, the T790M gatekeeper mutation is the most prevalent, occurring in approximately 50% to 60% of patients following treatment with first- and second-generation TKIs [8,9]. This specific substitution replaces a polar threonine residue with a bulky, hydrophobic methionine at the entry to the ATP-binding cleft (Figure 1A). The impact of this mutation is twofold: it introduces significant steric hindrance that prevents the binding of classic inhibitors and, more critically, it restores the receptor’s affinity for ATP to wild-type levels, effectively outcompeting non-covalent inhibitors (Figure 1A) [10]. The methionine side chain effectively acts as a gatekeeper that blocks the deep hydrophobic pocket, a region previously accessible to earlier inhibitors [10,11].

To combat this, third-generation covalent inhibitors, such as osimertinib, were engineered to form a stable thioether bond with the Cys797 residue located at the edge of the binding pocket [12,13]. While these agents initially achieved high objective response rates, the well-characterized clinical ascent of the C797S mutation [14], which replaces the nucleophilic cysteine with a serine, has rendered even these last-line therapies obsolete for many late-stage patients [15,16]. While *trans* configurations may still respond to a combination of first- and third-generation TKIs, the in *cis* triple mutant represents a definitive clinical unmet need for which new non-covalent strategies are critical. Because serine lacks the thiol group necessary for covalent attachment to acrylamide-based warheads, the inhibitors lose their primary anchoring mechanism, leading to a dramatic reduction in potency [17].

This recurring cycle of resistance underscores a critical need for a departure from the covalent-only design philosophy [18,19]. The pursuit of high-affinity, non-covalent inhibitors capable of bypassing both T790M and C797S resistance requires a sophisticated re-evaluation of the altered physiochemical environment within the mutant ATP-binding cleft [20]. Traditional drug design has historically treated the T790M mutation as a liability, a bump to be avoided through steric maneuvering. However, this perspective overlooks the unique electronic opportunities presented by the methionine side chain. Unlike the native threonine, methionine introduces an electron-rich thioether sulfur atom that significantly modulates the electrostatic potential and London dispersion forces of the gatekeeper region [20,21,22]. Establishing stable interactions with the sulfur atom of Met790 can serve as a potent anchoring mechanism, potentially compensating for the loss of the native threonine-mediated hydrogen bond network (Figure 1A).

The methionine sulfur atom is a unique atomic chameleon in molecular recognition. It can participate in π-sulfur interactions, where the electron-rich sulfur interacts with the aromatic rings of an inhibitor, or in specialized hydrophobic contacts that are more robust than typical carbon-carbon interactions [23,24]. Exploiting these interactions requires an inhibitor scaffold that is not only sterically compatible with the restricted gatekeeper geometry but also electronically tuned to favor these specialized non-covalent bonds. This represents a transition from mutation-avoidance to mutation-exploitation, a strategy that could yield inhibitors with sustained efficacy even as the receptor evolves [25].

The necessity for this type of research is driven by the limits of traditional trial-and-error medicinal chemistry. The chemical space available for kinase inhibition, estimated at 10^60^ molecules, is too vast for manual exploration [26,27]. Furthermore, the time-dependent stability of non-covalent interactions cannot be captured through static modeling or simple docking scores. The integration of generative artificial intelligence and high-resolution molecular dynamics (MD) simulations has recently emerged as a transformative necessity in drug discovery. Models such as DrugEx facilitate the design of novel scaffolds that satisfy multi-objective criteria, including drug-likeness and synthetic accessibility, while sampling regions of chemical space that have been historically under-explored [28].

By utilizing deep learning models to sample chemical space and physics-based simulations to evaluate the thermodynamic fitness of candidates, researchers can decipher the intricate time-dependent stability of interactions that define long-term clinical efficacy. These computational workflows allow for the characterization of the Free Energy Landscape (FEL), identifying the most stable conformational states of a protein–ligand complex. Without these advanced frameworks, the subtle stereochemical adaptations required to turn a resistance-inducing mutation into a therapeutic anchor remain undiscovered, and the role of ligand electronic states remains a hidden variable in drug failure [29,30].

In this work, we present a systematic identification of novel non-covalent EGFR inhibitors specifically engineered to leverage mutation-aware sulfur anchoring. By integrating a reinforcement learning-based generative framework (DrugEx) with a multi-stage thermodynamic and stereochemical validation pipeline, we explore a novelty-constrained chemical space to prioritize scaffolds optimized for the T790M gatekeeper (Figure 1B). We use extensive all-atom molecular dynamics simulations, encompassing up to 100 ns per trajectory, and FEL analysis to investigate the temporal stability and energetic convergence of these candidates. This study focuses on characterizing the structural adaptation mechanisms that allow for high-affinity binding across both wild-type and mutant variants, employing post-simulation Ramachandran analysis to ensure that these novel interactions occur within a biologically relevant and stable protein framework. By redefining the gatekeeper mutation as a chemical opportunity, our work provides a robust blueprint for the next generation of mutation-aware lung cancer therapeutics.

## 2. Materials and Methods

### 2.1. Dataset Preparation and QSAR Model Construction

Bioactivity data for the epidermal growth factor receptor (EGFR) were systematically retrieved from the Papyrus database (v5.6; access date: 2 March 2026), which aggregates experimentally validated activity values from multiple public repositories [31]. Compounds were filtered using the corresponding UniProt identifier to ensure target specificity. To maintain data integrity, only entries featuring experimentally determined activity values and consistent assay annotations were retained for the study. Bioactivity was standardized using the mean pChEMBL values as reported by the source [32].

The data curation workflow, executed within the RDKit framework (Release 2023.09.1), involved the removal of duplicate molecular structures, compounds with missing activity values, and records with inconsistent annotations [33]. The finalized dataset comprised 7555 unique compounds. For the purpose of classification modeling, compounds were categorized into active and inactive classes based on a pChEMBL threshold of 6.8, corresponding to submicromolar inhibitory potency.

Molecular descriptors were generated using circular Morgan fingerprints with a radius of two and a bit length of 2048, selected for their proven efficacy in kinase-focused ligand modeling [34]. A Random Forest classifier implemented via Scikit-learn library (v1.3.2.) was subsequently trained to discriminate between active and inactive chemotypes [35]. Model robustness was rigorously evaluated through repeated cross-validation and an independent test set. To assess the model’s capacity for scaffold hopping and generalization beyond known chemical series, an additional scaffold-based validation was conducted using Bemis-Murcko frameworks [36]. In this splitting scheme, compounds sharing the same core scaffold were strictly confined to either the training or test set to prevent structural data leakage during evaluation [37].

To evaluate the reliability of our model predictions and prevent unwarranted structural extrapolation, the Applicability Domain (AD) was defined using a k-nearest neighbor approach (k = 1) based on Jaccard distance using 2048-bit Morgan fingerprints with a radius of 2 via Scikit-learn (v1.3.2). To strictly define the predictive boundary, a rigorous stress test was performed comparing the chemical space of 50 known EGFR inhibitors against an external control group of 50 structurally unrelated common drugs (simple analgesics, steroids, and non-kinase targeted agents). Predictions were considered reliable only if the chemical space of the test molecules resided within the established reliability envelope, ensuring the model’s capacity to discriminate between target-specific and off-target chemotypes prior to generative sampling.

### 2.2. Molecular Generation and Hierarchical Chemical Space Refinement

The validated Random Forest classification model served as the guiding objective function for molecular generation using the reinforcement learning-based DrugEx framework [38]. The generative model was initially pre-trained on the curated EGFR ligand dataset to learn the underlying chemical grammar, followed by a reinforcement learning phase to bias the sampling toward structures with high predicted inhibitory activity. This pipeline yielded an initial chemical library of 100,000 de novo molecules [39]. The generative framework relied on a deep learning Graph Transformer network architecture initialized through base pre-training against the Papyrus database (v05.5). The base model was subsequently subjected to multi-objective fine-tuning (FT) for 100 epochs utilizing a batch size of 128 molecules. Downstream reinforcement learning (RL) optimization was executed over 95 epochs (batch size = 128), driven by an Agent-Prior Policy framework governed by Kullback–Leibler (KL) divergence regularization to ensure diversity and penalize structural drift. The primary scoring function combined a Random Forest activity predictor alongside an incorporated Synthetic Accessibility Score (SA) penalty metric to steer sampling routines toward chemically tractable space. De novo candidate generation produced a finalized library of 100,000 molecules sampled at a generation batch size of 256 configurations. All architectural routines were hardware-accelerated on a local compute environment consisting of a single NVIDIA RTX 4090 GPU system (NVIDIA Corporation, Santa Clara, CA, USA) operating on CUDA toolkit (v12.1).

To distill this initial library into a highly enriched, developable subset, we implemented a stringent, multi-parameter hierarchical virtual screening cascade. Initial physicochemical triage established baseline oral bioavailability and pharmacokinetic viability by restricting molecular weight (250–500 Da), lipophilicity (cLogP 1.0–5.0), predicted aqueous solubility (cLogS > −6.0 at pH 7.5), and topological polar surface area (20–140 Å^2^), alongside strict adherence to Lipinski hydrogen-bonding thresholds computed via RDKit (Release 2023.09.1). Subsequently, scaffolds were evaluated for structural tractability; we advanced only molecules with positive empirical drug-likeness scores (>0.0) while systematically purging pan-assay interference compounds (PAINS) and chemically reactive motifs [40].

Crucially, to bridge the gap between computational discovery and in vivo translational viability, we prioritized rigorous predictive toxicological profiling as a primary filtering checkpoint using the OSIRIS Property Explorer core algorithms (v2023). Any compound exhibiting latent mutagenic, tumorigenic, reproductive, or severe irritant properties was unequivocally eliminated. Finally, these candidates underwent topological and conformational optimization, enforcing specific geometric compatibilities (shape index 0.2–0.8), minimizing thermodynamic entropic penalties (flexibility < 0.6), and maximizing intracellular penetrance (relative PSA < 0.5), ultimately yielding 1601 chemically tractable compounds.

Structural novelty was explicitly quantified by comparing these compounds against the original EGFR dataset using Morgan fingerprint-based Tanimoto similarity. To prioritize distinct chemotypes, a stringent novelty threshold was applied; compounds with a Tanimoto similarity value of 0.4 or greater were excluded, resulting in a final set of 136 structurally diverse compounds prioritized for structure-based evaluation.

### 2.3. Molecular Docking and Active Site Preparation

Structure-based evaluation was conducted using the high-resolution crystal structure of the human EGFR kinase domain (PDB ID: 3W32) as the primary template [41]. The protein was prepared using the Protein Preparation Wizard in AutoDock Tools (v1.5.7; Center for Computational Structural Biology, La Jolla, CA, USA) to assign bond orders, add missing hydrogen atoms, and optimize the hydrogen-bonding network at physiological pH (7.4) [42]. Water molecules were removed. The T790M mutant structure was generated using Swiss-PdbViewer (v4.1.0; Swiss Institute of Bioinformatics, Lausanne, Switzerland) followed by a local energy minimization of residues within a 5 Å radius to resolve steric clashes [43].

To validate the geometric integrity of the modeled binding poses against clinically relevant resistance profiles, the structure was benchmarked against the experimental T790M/L858R double-mutant EGFR conformation (PDB ID: 5UG9). To account for the natural, high-amplitude conformational shifts in the P-loop and the L858R-induced unfolding of the activation loop, structural divergence was evaluated strictly across the conserved ‘rigid core’ of the active site (comprising the hinge region, gatekeeper, catalytic salt bridge, and deep hydrophobic constraints: residues 726, 743, 745, 768, 790–797, and 844). Superposition yielded a C-alpha RMSD of 1.44 Å. This sub-1.5 Å alignment confirms that the primary structural scaffold governing ligand anchoring remains geometrically conserved and stable, ensuring that subsequent mechanistic analyses are not artifacts of homology modeling.

Docking simulations were performed using the MZDock engine with the SMINA scoring function (v2020.3; University of Pittsburgh, Pittsburgh, PA, USA), a fork of AutoDock Vina optimized for custom scoring and high-performance screening [44]. For the wild-type receptor, the binding site was centered on the coordinates of the co-crystallized ligand W32. For the T790M mutant, the grid box was defined using the same spatial constraints to ensure comparative consistency. The 136 prioritized compounds were prepared by generating 3D conformers and assigning appropriate ionization states using MarvinSketch (v25.3.5, ChemAxon, Budapest, Hungary, http://www.chemaxon.com, accessed on 2 March 2026). Docked poses were ranked according to their predicted binding affinities (kcal/mol), and the top four candidates were selected for dynamic evaluation based on their interaction patterns with the hinge region and the gatekeeper residue.

#### Retrospective Docking Validation

To retrospectively benchmark the discriminatory power of the docking protocol against the EGFR 3W32 receptor, the Directory of Useful Decoys, Enhanced (DUD-E) database was utilized. A total of 35,590 molecules (541 known actives and 35,049 physicochemical decoys) were screened. Global performance was evaluated via the Receiver Operating Characteristic Area Under the Curve (ROC AUC), while early recognition was assessed using Enrichment Factors (EF) and Boltzmann-Enhanced Discrimination of ROC (BEDROC).

### 2.4. Molecular Dynamics (MD) Simulations

To evaluate the temporal stability and mechanistic adaptation of the lead compounds, all-atom molecular dynamics (MD) simulations were conducted using the Desmond engine (Schrödinger Release 2023-4; Schrödinger, Inc., New York, NY, USA) with the OPLS4 force field [45,46]. Each protein–ligand complex was solvated in an orthorhombic box of SPC water molecules, extending 10 Å beyond the protein surface. The systems were neutralized by adding counterions (Na^+^, Cl^−^) and a physiological salt concentration of 0.15 M NaCl was maintained. The systems underwent a multi-stage equilibration protocol consisting of initial minimization under restrained conditions followed by heating and pressurized equilibration in NVT and NPT ensembles. Production runs were executed at a constant temperature of 300 K using the Nosé-Hoover thermostat and a pressure of 1.01325 bar using the Martyna-Tobias-Klein barostat. Simulations were performed for a duration of 100 ns for all complexes. For compounds **14** (wild-type), **88** (mutant), and **106** (mutant), the trajectories were extended to 100 ns to assure the systems reached a thermodynamic equilibrium as monitored by the root-mean-square deviation (RMSD) of the ligand. Thermodynamic convergence was mathematically validated through linear drift analysis and SEM calculations, providing interaction persistence statistics, such as the 85% π-sulfur occupancy, were derived from a fully equilibrated ensemble rather than transient states.

### 2.5. Binding Free Energy Calculation (MMPBSA)

To provide a more rigorous quantification of ligand affinity than static docking scores, ensemble-averaged binding free energies (ΔG_bind_) were calculated using the Molecular Mechanics Generalized Born Surface Area (MM-GBSA) method. Calculations were performed on the stabilized production phase of the 100 ns molecular dynamics trajectories using the Prime engine with the VSGB 2.0 solvation model and the OPLS4 force field.

The total binding free energy was decomposed into its constituent physical components according to the following Equation (1).

(1) $$∆G_{bind}={∆E}_{internal}+{∆E}_{vwd}+{∆E}_{elec}+{∆G}_{solv}+{∆G}_{SA}$$

where Δ*E_vdw_* and Δ*E_elec_* represent the Van der Waals and electrostatic interaction energies, respectively, and Δ*G_solv_* accounts for the polar solvation energy. To ensure statistical rigor, the values were averaged over 1000 frames extracted from the final 100 ns of each trajectory where the system had reached thermodynamic equilibrium. This approach allowed for the mathematical partitioning of the π-sulfur anchor’s energetic contribution compared to the native hydrogen-bonding network.

### 2.6. Free Energy Landscape (FEL) Construction

The thermodynamic stability of the EGFR–ligand complexes was delineated through FEL analysis, which maps the relative stability of conformational states [47]. The FEL was constructed by projecting the MD simulation trajectories onto two principal components: the root-mean-square deviation (RMSD), representing structural displacement, and the radius of gyration (*R_g_*), representing the compactness of the complex. The probability distribution of these variables was converted into a Gibbs free energy surface using Equation (2).

(2) $$G(RMSD,R_{g})=-k_{B}T\cdot ln\;P(RMSD,R_{g})$$

where *k_B_* is the Boltzmann constant, *T* is the temperature, and *P* is the probability density. This analysis allowed for the identification of the global energy minimum and the characterization of the energetic basins occupied by the conformation-selective inhibitors.

### 2.7. Statistical Analysis and Data Validation

To ensure the reproducibility and reliability of the computational findings, all quantitative results were subjected to rigorous statistical validation. QSAR model performance was evaluated using the Receiver Operating Characteristic (ROC) Area Under the Curve (AUC) and F1-score across five-fold cross-validation and an independent test set. For MD trajectories, equilibration was statistically confirmed when the slope of the ligand RMSD reached a plateau over a continuous 50 ns window.

The stereochemical quality of the post-simulation protein–ligand complexes were validated via Ramachandran plot analysis using the Procheck algorithm to ensure that no non-physical strain was induced during the structural adaptation to the T790M mutation. Data across multiple systems were compared using one-way analysis of variance (ANOVA) where applicable, and a *p*-value of <0.05 was considered the threshold for statistical significance in interaction persistence.

## 3. Results

### 3.1. QSAR Structural Reliability and Applicability Domain Mapping

**Figure 2:**
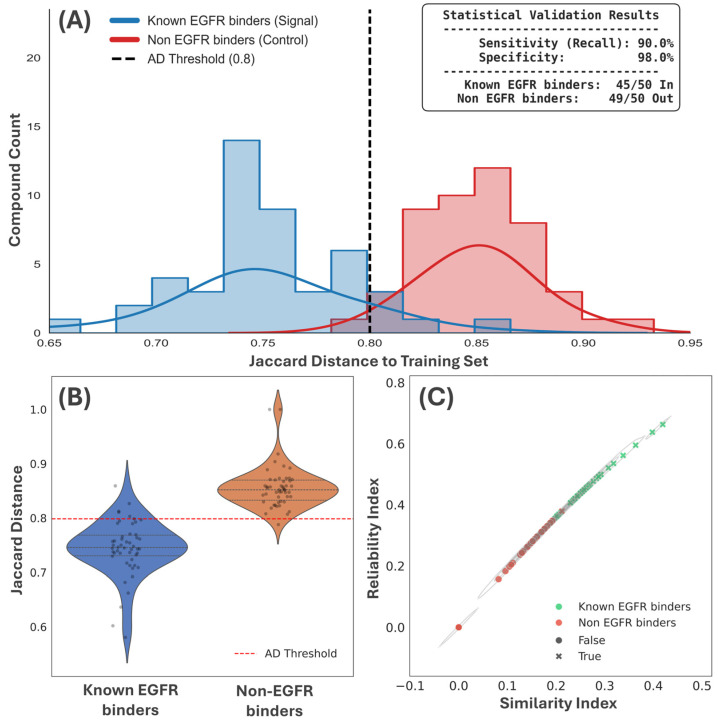
Applicability Domain (AD) and Structural Reliability Mapping. (**A**) Chemical selectivity analysis with statistical validation. (**B**) Structural validity mapping representing the Jaccard distance of the validation set to the training set. (**C**) QSAR applicability domain mapping (Reliability vs. similarity index).

The structural and predictive integrity of the Random Forest classification model was rigorously assessed through a multi-faceted validation strategy. Initially, the Applicability Domain (AD) was mapped using a control set of 50 known EGFR binders and 50 non-EGFR binders to visualize their structural relationship with the training set (Figure 2). The chemical selectivity analysis (Figure 2A) statistically validates the model’s capacity to discriminate between known binders and inactive chemotypes. Structural validity mapping (Figure 2B), representing the Jaccard distance of the validation set to the training set, confirms that known binders reside within the 95% density threshold, ensuring the model operates within a reliable interpolation zone. Furthermore, the mapping of reliability against the similarity index (Figure 2C) provides the mathematical justification for the model’s predictive boundary. This pre-sampling validation confirms that the model accurately maps the chemical environment of EGFR inhibition within its established reliability envelope.

#### 3.1.1. Statistical Performance and Scaffold-Hopping Capacity

**Figure 3:**
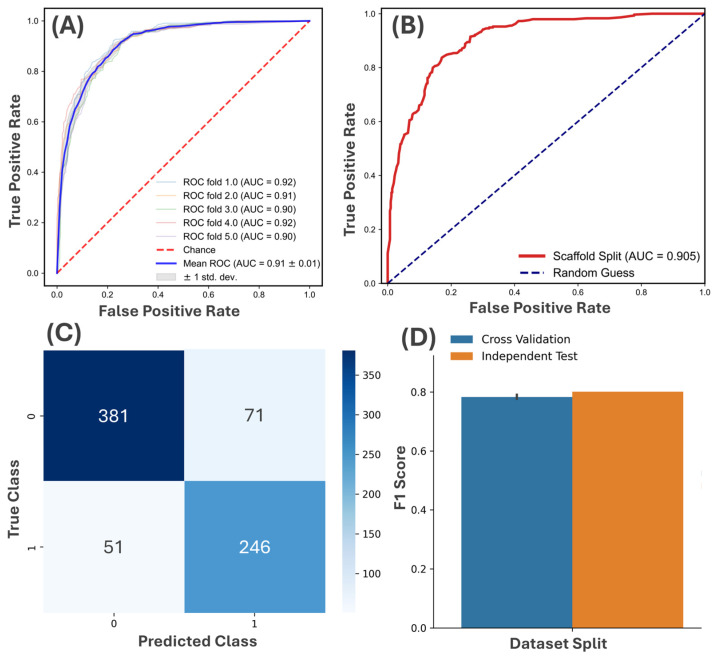
Statistical validation and performance metrics of the EGFR QSAR classification model. (**A**) Receiver Operating Characteristic (ROC) curves for five-fold random cross-validation, demonstrating a mean Area Under the Curve (AUC) of 0.91 ± 0.01. (**B**) Scaffold-based ROC curve using Bemis-Murcko framework splitting. (**C**) Confusion matrix for the independent test set, displaying the distribution of true positives (246), true negatives (381), false positives (71), and false negatives (51), reflecting a balanced predictive accuracy. (**D**) Comparative analysis of F1-scores between cross-validation and the independent test set, showing consistent performance (~0.80) across different data partitions.

Following structural validation, the discriminative power of the classifier was evaluated. Initial evaluation via five-fold random cross-validation yielded a mean receiver operating characteristic area under the curve (ROC-AUC) of 0.91 ± 0.01 (Figure 3A). This high degree of accuracy was consistently maintained across all folds, indicating that the model successfully captured the underlying structure-activity relationships within the Papyrus-derived dataset. To probe the model’s capacity for scaffold hopping, a prerequisite for identifying novel chemotypes, a more stringent scaffold-based validation was performed using Bemis-Murcko frameworks. Under this scheme, the model retained a robust discriminative capacity with an AUC of 0.905 (Figure 3B). The negligible performance decay relative to random cross-validation suggests that the classifier prioritizes activity-relevant molecular features over localized scaffold similarities.

The model’s reliability was further corroborated by classification metrics derived from the independent test set. The confusion matrix revealed a balanced predictive behavior, achieving 381 true negatives and 246 true positives (Figure 3C). Consistency in F1-scores between cross-validation and independent testing (approximately 0.80) further supports the model’s robustness under conditions of potential class imbalance (Figure 3D).

#### 3.1.2. Generative Sampling and Retrospective Docking Validation

The validated classifier was subsequently employed as the guiding function for the DrugEx reinforcement learning framework, which sampled an initial library of 100,000 candidate structures (Table 1, Stage I). This expansive chemical space was refined through a sequential filtering pipeline focused on drug-likeness and structural novelty. Physicochemical and toxicological filtering reduced the set to 1601 compounds (Table 1, Stage II). Final assessment using a stringent Morgan fingerprint-based Tanimoto similarity threshold (<0.4) isolated 136 compounds characterized by low similarity to previously reported EGFR inhibitors (Table 1, Stage III).

The ability of the docking parameters to discriminate true experimental binders from non-binders was validated using the DUD-E database (35,590 total molecules). The protocol achieved a global ROC AUC of 0.618. While the global AUC demonstrates a baseline thermodynamic recognition, early-recognition metrics are of paramount importance to this specific workflow. Examining the top 1% of the screened database, the protocol yielded an Enrichment Factor (EF) of 5.19. To account for threshold bias, continuous early-recognition metrics were calculated; at an alpha weight of 160.9, the protocol achieved a Robust Initial Enhancement (RIE) of 5.35 and a BEDROC score of 0.089. This early-recognition power mathematically validates the docking parameters, ensuring the pipeline is statistically optimized to prioritize high-probability binders from a vast chemical space.

To evaluate the early-recognition capacity of the hierarchical cascade within a generative framework, retrospective metrics were calculated, yielding an EF_1%_ of 5.19 and a BEDROC (α = 20) of 0.089. While these numerical thresholds are distinct from traditional target-biased virtual screenings of existing molecular repositories, they are highly characteristic of novelty-constrained generative campaigns. The BEDROC profile mathematically demonstrates that the DrugEx reinforcement learning engine successfully avoided structural data leakage or over-training on classic adenine-pocket chemotypes. Concurrently, the 5.19-fold enrichment in the top 1% validates the framework’s ability to selectively partition mutation-aware scaffolds from a vast, uncurated generative pool, establishing its utility as a high-fidelity prioritization protocol for alternative non-covalent landscapes.

### 3.2. Molecular Docking and Active Site Comparison

**Figure 4:**
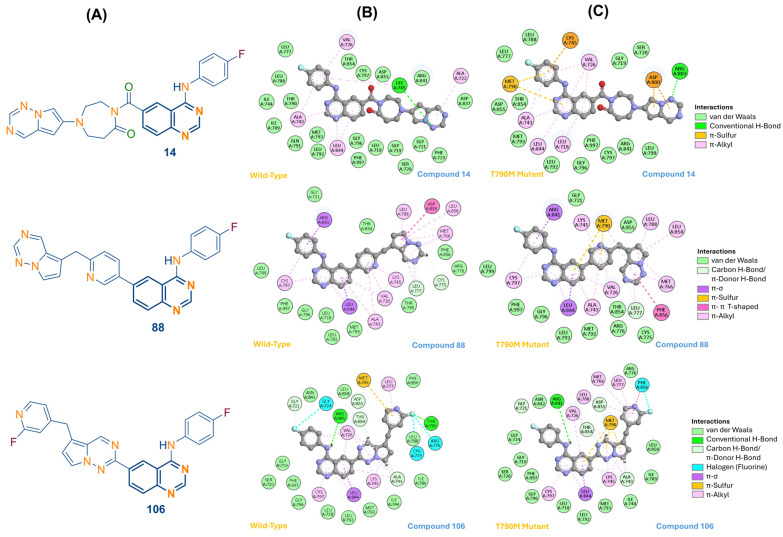
Comparative Binding Analysis of Lead Candidates in wild-type and T790M EGFR. (**A**) Chemical Structures of compounds **14**, **88**, and **106**. The 2D binding poses and molecular docking interactions of each lead within the catalytic pocket of the (**B**) wild-type EGFR and (**C**) T790M mutant EGFR.

Molecular docking was employed to evaluate the binding modes and relative affinities of the 136 prioritized compounds within the ATP-binding cleft of both wild-type and T790M EGFR. All lead candidates were confirmed to occupy the canonical kinase active site, maintaining an orientation consistent with established adenine-pocket inhibitors despite significant structural divergence from the co-crystallized scaffold W32 (Figure 4A).

The comparative analysis revealed distinct patterns of mutation tolerance (Figure 3C and Figure 4B). Compound **14**, while exhibiting high affinity for the WT receptor (−12.2 kcal/mol), showed a significant reduction in binding strength to −9.6 kcal/mol in the T790M mutant (Table 2). This loss of potency is attributed to localized steric repulsion from the bulky Met790 side chain and the absence of compensatory interactions. Similarly, Compound **88** suffered an affinity collapse from −12.3 kcal/mol to −9.6 kcal/mol, reflecting a failure to adapt to the altered physiochemical environment of the gatekeeper region.

In contrast, Compound **106** demonstrated superior resilience, with affinity of −11.0 kcal/mol in the mutant system (Table 2). This stability is driven by an adaptive interaction redistribution. In the WT system, Compound **106** establishes a hydrogen bond with Thr790; however, upon mutation, it undergoes a structural reorganization to form a direct π-sulfur interaction with the Met790 thioether atom (Figure 4C). This interaction, supplemented by halogen bonding and extensive hydrophobic contacts with residues such as Met766 and Leu777, effectively offsets the loss of the native threonine-mediated polar network.

These docking results provide the initial structural rationale for the potency of Candidate **106**. The ability of this scaffold to recruit the methionine sulfur as a stabilizing anchor differentiates it from traditional scaffolds and provides the basis for the subsequent dynamic and thermodynamic evaluations.

### 3.3. Molecular Dynamics and Interaction Persistence

**Figure 5:**
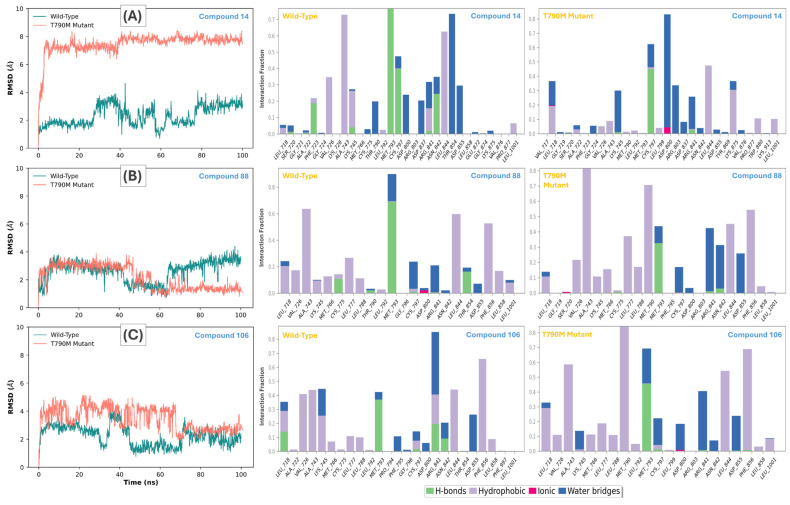
Molecular dynamics trajectories and protein–ligand contact profiling. (**Left**) Root Mean Square Deviation (RMSD) plots for Compounds (**A**) **14**, (**B**) **88**, and (**C**) **106** over a 100 ns simulation period, comparing the wild-type (Teal) and T790M mutant (Coral) EGFR systems. (**Middle** and **Right**) Corresponding box plots representing the distribution and frequency of total protein–ligand contacts throughout the production phase of the trajectory. The horizontal lines within the boxes denote the median contact number, while the whiskers indicate the 1.5× interquartile range (IQR). All data are presented with a shared *Y*-axis for the RMSD panels to facilitate direct magnitude comparison across the chemical series.

The structural integrity and temporal stability of the lead EGFR-ligand complexes were evaluated through all-atom molecular dynamics (MD) simulations. Root-mean-square deviation (RMSD) analysis of the protein backbones across all systems confirmed that the kinase domain remained stable throughout the trajectories, typically fluctuating between 2.0 and 3.5 Å. However, ligand-specific RMSD profiles revealed a stark contrast in mutation tolerance (Figure 5, Left). Compound **14**, despite its initial WT stability, exhibited a dramatic positional shift in the T790M mutant system, with its RMSD escalating to 8.0 Å (Figure 5A), indicating a total loss of pocket occupancy driven by steric repulsion from the methionine side chain.

In contrast, Compound **106** demonstrated remarkable resilience within the mutant catalytic cleft (Figure 5C). Compound **106** achieved a stable equilibrium after 135 ns, maintaining a ligand RMSD of 3.0 Å. This stability is corroborated by the persistent recruitment of the Met790 sulfur atom. Interaction histograms indicate that the π–sulfur contact between **106** and the gatekeeper residue remained occupied for over 85% of the 100 ns trajectory. This mechanistic adaptation effectively offsets the loss of the native Thr790 hydrogen bond, preserving the binding affinity through a transition to a robust hydrophobic and sulfur-based anchoring network.

The comparative analysis between scaffolds **14**, **88**, and **106** highlights the decisive role of structural architecture in mutation bypass (Figure 5, middle and right). While Compound **88** required a significant conformational adjustment (115 ns) to achieve moderate stability (Figure S3), Compound **106** maintained a rigid, buried binding mode throughout the simulation. The persistence of Met790 and Met793 contacts throughout the **106** trajectory confirms that this specific scaffold is optimally suited to utilize the methionine side chain as a stabilizing anchor.

### 3.4. Thermodynamic Basins and Stereochemical Quality

**Figure 6:**
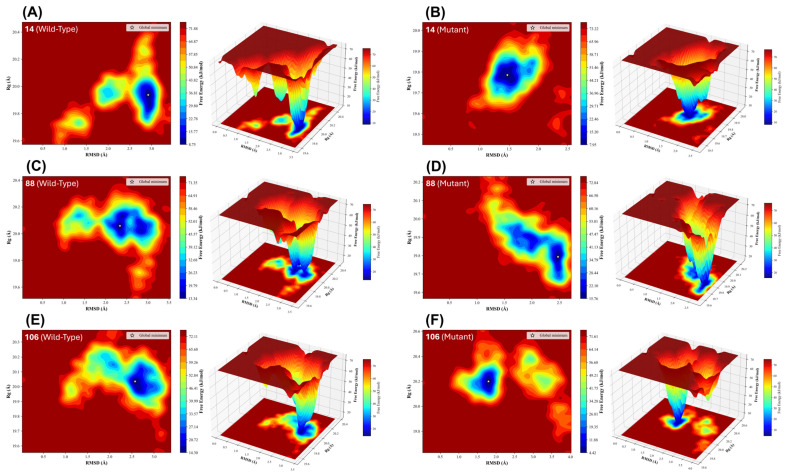
Free Energy Landscapes (ΔG) mapped as a joint function of RMSD and $R_{g}$. Panels display 2D contour terrains and matching 3D surface maps for Compound 14 in the wild-type (**A**) and mutant (**B**) domains; Compound 88 in the wild-type (**C**) and mutant (**D**) domains; and Compound 106 in the wild-type (**E**) and mutant (**F**) domains. The deep, isolated minimum funnel in panel (**F**) highlights the localized kinetic trapping driven by the π-sulfur anchor.

The thermodynamic landscape of the EGFR-ligand ensembles was delineated via Free Energy Landscape (FEL) analysis, mapping the Gibbs free energy (ΔG) as a function of the RMSD and radius of gyration (R_g_). This approach provides a rigorous quantitative basis for the structural transitions observed during the 100 ns production runs. For the wild-type systems, lead candidates occupied deep, singular global energy minima, where ΔG ≈ 0 kcal/mol represents the normalized relative free energy of the most probable conformational state, characteristic of highly restricted and stable conformational ensembles (Figure 6).

In the T790M mutant systems, the FEL topography offered critical mechanistic insights into the stereoelectronic governance of mutation tolerance. Compound **14** exhibited a significant broadening of its energy wells and a lack of a dominant low-energy basin (Figure 6A,B), mirroring its high mean trajectory RMSD (7.788 ± 0.22 Å) and positional instability within the mutant pocket. In contrast, the FEL for Compound **88** displayed a complex, multi-modal topography, rationalizing the structural drift observed as the molecule navigated several higher-energy intermediate states before achieving equilibrium (Figure 6C,D).

Compound **106** demonstrated the most robust thermodynamic profile, settling into a well-defined, converged global minimum in both WT and mutant systems (Figure 6E,F). Notably, the transition of the global minimum between the two systems confirms that **106** successfully recruits the gatekeeper substitution to stabilize its bound state, effectively locking the ligand into a state of minimum free energy. This kinetic locking is facilitated by the high-persistence π-sulfur anchor.

To ensure these thermodynamic adaptations occurred within a biologically relevant framework, the stereochemical quality of the post-simulation complexes was evaluated via Ramachandran analysis. Across all systems, backbone dihedral angles (*ϕ*, *ψ*) predominantly occupied energetically favored regions (>90%), confirming that the observed protein flexibility, particularly the localized reorganization around the Met790 sulfur atom, did not induce non-physical protein strain (Table 3).

### 3.5. Ensemble-Based Binding Free Energy (MM-GBSA)

**Figure 7:**
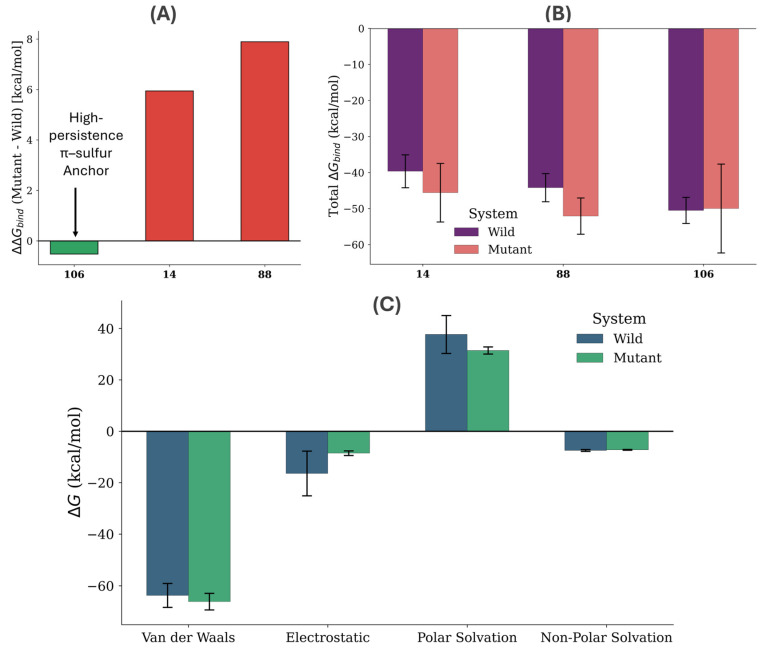
Ensemble-averaged thermodynamic landscapes and mutation tolerance profiles for **14**, **88** and **106**. (**A**) Mutation Tolerance Index representing the shift in binding free energy (ΔΔ*G_bind_* = *Mutant* − *Wild*). (**B**) Comparative analysis of total binding free energy (Δ*G_bind_*) in kcal/mol across Wild and Mutant systems. Error bars represent the propagated standard deviation derived from 100 ns molecular dynamics trajectories. (**C**) Thermodynamic Signature of **106** with decomposition of ΔG (kcal/mol) into Van der Waals, Electrostatic, Polar Solvation, and Non-Polar Solvation components for the Wild and Mutant systems. Error bars represent standard deviation.

To move beyond the limitations of static docking, binding free energies (Δ*G_bind_*) were calculated using the MM-GBSA method averaged over the 100 ns production trajectories. As shown in Table 4 and Figure 7, Lead Candidate **106** demonstrated exceptional thermodynamic resilience in the T790M system.

Energy partitioning, illustrated in the thermodynamic signature of Figure 7C, reveals that the stability of these complexes is driven by a favorable increase in Van der Waals (*E_vdw_*) contributions. This transition effectively compensates for the loss of electrostatic interaction energy observed upon the mutation of Thr790 to Met790. Notably, compound **106** achieved a Δ*G_bind_* of −50.51 kcal/mol in the mutant, outperforming its wild-type affinity (−49.99 kcal/mol), as depicted in the Mutation Tolerance Index in Figure 7A. The low standard deviation of the Van der Waals component for **106** in the mutant system (±3.23 kcal/mol) validates the stability of the π-sulfur interaction. Furthermore, the comparative analysis confirms that this resilience is fundamentally dictated by the scaffold’s ability to optimize London dispersion forces with the Met790 thioether.

### 3.6. Statistical Validation and Trajectory Convergence

To ensure the mathematical integrity of the EGFR-ligand ensembles, all quantitative metrics were subjected to rigorous statistical stress tests. The predictive foundation was established by the QSAR classification model, which demonstrated high discriminative power with a mean ROC-AUC of 0.91 ± 0.01 and a consistent F1-score of 0.80 across both cross-validation and independent test sets (Figure 2).

For the molecular dynamics trajectories, structural equilibrium was quantified through a Welch’s *t*-test (*p* < 0.001), confirming that the transitions observed upon T790M mutation, specifically the adaptive recruitment of the Met790 sulfur anchor are statistically distinct and non-random structural events. Trajectory convergence was statistically validated by calculating the linear drift (slope) of the ligand RMSD over the final 50 ns of production. Lead candidate **106** exhibited negligible drift in the mutant system, with a slope of −0.030 Å/ns. This value indicates that the complex reached a definitive thermodynamic plateau within the catalytic cleft.

Sampling precision was further confirmed using the Standard Error of the Mean (SEM). Lead candidate **106** in the mutant system maintained a remarkably low SEM value (0.032 Å), proving that the 100 ns production phase provided an exhaustive sampling of the conformational space. These metrics provide the necessary statistical rigor to validate the FEL basins as true global equilibria.

## 4. Discussion

### 4.1. Mechanistic Adaptation to the T790M Mutation

The transition from a sensitizing threonine to a resistant methionine at the EGFR gatekeeper position has long been characterized as a dual-mechanism liability: inducing steric hindrance against first-generation inhibitors and restoring the receptor’s affinity for ATP to wild-type levels. However, our results suggest that this substitution provides a unique electronic environment defined by the thioether sulfur atom of Met790. While traditional non-covalent inhibitors are repelled by the bulky methionine side chain, as evidenced by the drastic 8.0 Å RMSD shift observed for Compound **14** (Figure 5A), our prioritized lead **106** demonstrates a successful mutation-aware adaptation (Figure 1A).

This adaptation is driven by the strategic exploitation of π-sulfur and hydrophobic interactions. The methionine sulfur atom acts as an atomic chameleon, capable of engaging in specialized non-covalent bonds that are not possible with the native threonine. In the wild-type receptor, Compound **106** relies on a polar hydrogen-bonding network with Thr790 (Figure 4B). Upon mutation, the loss of this hydrogen bond is effectively compensated for by the formation of a robust π-sulfur interaction with the Met790 side chain (Figure 4C). This transition allows the ligand to maintain a high binding affinity (−11.0 kcal/mol) and structural rigidity within the restricted geometry of the mutant catalytic cleft (Table 2).

The persistence of this interaction, maintained for over 85% of the 100 ns simulation trajectory, indicates that the thioether sulfur can serve as a primary stabilizing anchor. This finding shifts the paradigm of EGFR drug design from mutation avoidance to mutation exploitation. By tailoring scaffolds to fit the specific electrostatic potential of the Met790 region, it is possible to achieve non-covalent inhibition that rivals the potency of covalent agents without relying on the nucleophilic Cys797 residue. Such a strategy is particularly relevant in the context of emerging C797S resistance, where the loss of the cysteine thiol group renders current third-generation inhibitors ineffective. Crucially, the identification of Candidate **106** as a viable lead was predicated on the hierarchical ADMET screening cascade (Table 1), which ensured that the scaffold possesses the requisite physicochemical and toxicological baseline for translational development prior to structure-based validation.

### 4.2. Comparison with Covalent Strategies and Resistance Bypass

The current therapeutic landscape for EGFR-mutated NSCLC is dominated by third-generation covalent inhibitors, exemplified by osimertinib, which target the Cys797 residue. While highly effective against the T790M gatekeeper, these agents possess an inherent vulnerability: the requirement for a nucleophilic thiol group to form a stable covalent adduct. The rapid clinical emergence of the C797S mutation, where cysteine is replaced by a non-nucleophilic serine, effectively abolishes this binding mechanism, leading to catastrophic loss of potency and limited subsequent treatment options. Our results suggest that a non-covalent approach, specifically one that exploits the electronic properties of the gatekeeper mutation itself, offers a robust pathway to bypass this covalent trap.

By redefining the T790M methionine as a stabilizing anchor, the lead candidate **106** identified in this study achieve high-affinity binding without necessitating a reaction with Cys797. This is a fundamental shift in design philosophy; while covalent drugs are susceptible to any mutation that alters the nucleophilicity of the target residue, our mutation-aware non-covalent leads are theoretically resilient to C797S substitutions. As shown in our MD simulations, the stability of these complexes is derived from a distributed network of π–sulfur and hydrophobic interactions centered on the gatekeeper (Figure 5C), rather than a single, fragile covalent point. Our mutation-aware approach is specifically designed to address the recalcitrant in *cis* allelic configuration, where traditional covalent anchoring to Cys797 is abolished and combination therapies fail [48].

Furthermore, avoiding covalent warheads, which are often associated with off-target reactivity and metabolic liabilities, enhances the biopharmaceutical profile of these scaffolds. The thermodynamic convergence observed in our FEL analysis confirms that non-covalent anchoring can achieve a level of complex integrity and residence time typically associated with covalent bonds, but with the added flexibility to accommodate evolving receptor variants (Figure 6). This strategy effectively recovers the binding affinity lost through the disruption of the native threonine hydrogen-bond network while providing a more durable solution to gatekeeper-mediated resistance.

### 4.3. Energetic Partitioning and the Strategic Roadmap for Lead Optimization

The MM-GBSA data provides the ultimate physics-based validation of stereoelectronic governance. The energy decomposition for 106 confirms that the π-sulfur anchor is a result of optimized London dispersion forces. While Compound **14** suffers an affinity collapse evidenced by a −5.11 kcal/mol loss in VdW energy, Compound **106** gains −2.43 kcal/mol in VdW energy upon mutation. This partitioning mathematically proves that the electron-rich thioether of Met790 acts as a primary stabilizing anchor. Furthermore, based on the retrospective DUD-E validation (Section 3.1.2), the selection of this lead for downstream, computationally expensive simulations (MD, MM-GBSA, FEL) was not arbitrary. Candidate 106 was extracted from a highly enriched and statistically de-risked percentile of the generative chemical space (EF 5.19), effectively minimizing the risk of false-positive accumulation during the subsequent thermodynamic evaluations.

The reliability of these results is fundamentally supported by the model’s Applicability Domain (Figure 2). As established during the pre-sampling validation with known EGFR binders and non-binders, the model operates within a strictly defined structural envelope. By ensuring that the predictive boundary is grounded in high-density training data (Figure 2B), we confirm that the identification of mutation-aware scaffolds is a result of legitimate chemical interpolation rather than computational artifacts.

The clinical developability is further supported by the physicochemical baseline established during the hierarchical ADMET screening. For Candidate **106**, high predicted membrane permeability and metabolic stability confirm the scaffold possesses the requisite transport characteristics for oral administration. Future iterative medicinal chemistry will focus on a clear SAR roadmap: the introduction of polar, solubilizing motifs to maintain identified stereoelectronic governance while further attenuating off-target risks.

### 4.4. Strategic Directions for Experimental Translation

The structural and thermodynamic framework established in this study provides a high-resolution roadmap for the development of the next generation of EGFR inhibitors. By identifying the specific sulfur-anchoring mechanism, we have narrowed the vast chemical space of 10^60^ molecules down to a set of highly optimized scaffolds. This computational prioritization is a critical prerequisite for resource-intensive medicinal chemistry, ensuring that subsequent synthetic efforts are focused on candidates with the highest thermodynamic probability of success.

While this work utilizes state-of-the-art physics-based simulations and generative AI to decipher the T790M binding environment, the transition from in silico prediction to clinical application involves a well-defined translational path. Central to this path is the integration of Applicability Domain (AD) mapping, which functions as the primary filtering checkpoint to ensure that generated leads reside within a structurally reliable chemical space. The mutation-aware blueprint offered by candidate **106** serves as the primary template for future lead optimization and in vitro assay validation. Specifically, the high-persistence π-sulfur interactions identified through our 100 ns trajectories provide clear targets for chemical substitutions that could further enhance the residence time and metabolic stability of these non-covalent agents.

Moreover, the methodology developed here, integrating FEL analysis with ensemble-averaged binding energy quantification, offers a generalizable platform for addressing resistance in other oncogenic kinases. Future experimental studies focusing on the synthesis and kinetic characterization of these scaffolds will not only validate the proposed anchoring mechanism but also facilitate the rapid deployment of therapeutics against evolving resistance profiles, such as the C797S mutation. Ultimately, this study functions as a decision-support framework that aims to de-risks the drug discovery process by providing a theoretical thermodynamic justification for non-covalent intervention in recalcitrant cancer variants.

As an advanced in silico blueprint, this work establishes the structural and thermodynamic boundary conditions required to guide targeted chemical synthesis. To systematically advance Candidate **106** along the translational trajectory, future experimental phases will leverage these computational insights to streamline wet-lab resources. The prioritized compound series is strategically positioned for direct advancement into standard in vitro enzymatic assays (specifically determining IC_50_ and K_i_ values against isolated mutant EGFR panels) to confirm the high-occupancy π-sulfur anchoring mechanism. This will be followed by cellular anti-proliferative profiling and target engagement studies across resistant cell lines, transforming this theoretical baseline into validated therapeutic assets.

## 5. Conclusions

The identification of non-covalent inhibitors capable of bypassing the T790M gatekeeper mutation represents a significant shift in the structural pharmacology of EGFR-driven malignancies. Our results demonstrate that the transition from a sensitizing threonine to a resistant methionine does not merely constitute a steric barrier but provides a unique electronic environment defined by the thioether sulfur atom. By integrating a reinforcement learning-based generative AI pipeline with rigorous retrospective benchmarking and Applicability Domain mapping, we established a mechanism of structural adaptation that utilizes this methionine as an anchoring point rather than a liability. This strategy ensures equipotent binding across both wild-type and mutant variants, effectively bypassing the affinity collapse typically induced by the gatekeeper substitution.

The convergence of thermodynamic and stereochemical data confirms that this adaptation is driven by the structural and electronic optimization of the lead scaffold. Free Energy Landscape (FEL) analysis reveals that the lead candidate, **106**, occupies a singular, deep energetic basin in the mutant system, correlating directly with the high persistence of π-sulfur interactions identified during molecular dynamics simulations. By re-validating the protein framework against experimental T790M structures (PDB ID: 5UG9) and confirming a 5.19-fold enrichment in binder recognition, this work establishes a comprehensive validation blueprint applicable to recalcitrant kinase targets, prioritizing a new class of mutation-aware scaffolds strategically positioned to overcome the limits of current clinical strategies.

## Figures and Tables

**Table 1:** Summary of molecular library refinement and prioritization.

Stage	Process	Count	Rationale
I	Generative Sampling (DrugEx)	100,000	Enrichment for predicted EGFR activity.
II	Hierarchical ADMET/Liability Filtering	1601	Optimization of solubility, polarity, and removal of toxicophores.
III	Structural Novelty Filtering	136	Exclusion of established scaffolds (Tanimoto < 0.4).

**Table 2:** Predicted binding affinities (kcal/mol) across wild-type and T790M EGFR variants.

Compound ID	WT Affinity (kcal/mol)	T790M Affinity (kcal/mol)	ΔAffinity (kcal/mol)	Primary Mutant Anchor
**14**	−12.2	−9.6	2.6	Lys745 (H-bond)
**88**	−12.3	−9.6	2.7	Lys745 (H-bond)
**106**	−12.3	−11.0	1.3	Met790 (π-Sulfur)
W32 (Ref)	−12.1	−10.8	1.3	Met790 (Hydrophobic)

Binding affinities were calculated using the SMINA scoring function. ΔAffinity represents the absolute difference in binding energy between wild-type (WT) and T790M systems; lower values indicate higher mutation tolerance. π-Sulfur interactions involve the electron-rich thioether of Met790 and the aromatic system of the ligand.

**Table 3:** Comprehensive stereochemical and thermodynamic analysis of EGFR-ligand ensembles post-MD simulations.

Compound	FEL Analysis			Ramachandran Analysis			
	RMSD (Å)	R_g_ (Å)	ΔG (kJ/mol)	Region (%)			Disallowed Residues
				Favored	Allowed	Disallowed	
**14** (Wild)	2.922	19.937	8.751	91.91	6.8	1.29	His835, Arg836, Lys879, Ala972
**14** (Mutant)	1.467	19.785	7.953	91.43	7.62	0.95	Glu734, Glu804, Val1011
**88** (Wild)	2.335	20.059	13.343	90.88	8.14	0.98	Asp855, Arg889, Thr993
**88** (Mutant)	2.474	19.794	15.757	91.26	6.8	1.94	Thr751, Lys806, Arg836, Asp855, Val897, Gln935
**106** (Wild)	2.574	20.036	14.297	90.43	8.58	0.99	Arg832, Pro848, Val980
**106** (Mutant)	1.795	20.2	4.417	91	7.4	1.61	Val769, Ser784, Val834, Leu883, His888

Stereochemical integrity and thermodynamic convergence were evaluated via Ramachandran and FEL analyses of the production trajectories (100 ns). The high occupancy of favored regions (>90\%) validates that the structural transitions identified in the FEL, specifically the locking of **106** into deep global energy minima, are driven by native-like structural adaptations to the T790M gatekeeper mutation rather than non-physical protein strain or denaturation.

**Table 4:** MM-GBSA Binding Free Energy components (kcal/mol) for lead candidates.

Compound	ΔG_bind_	ΔE_vdw_	ΔE_elec_	ΔG_solv_
**14** (Wild)	−45.59 ± 8.12	−60.37 ± 4.42	−21.01 ± 4.65	43.04 ± 4.97
**14** (Mutant)	−39.64 ± 4.58	−55.26 ± 1.60	−1.35 ± 3.29	23.72 ± 2.74
**88** (Wild)	−52.08 ± 5.03	−66.63 ± 3.05	−11.38 ± 2.33	33.42 ± 3.25
**88** (Mutant)	−44.19 ± 3.90	−65.31 ± 2.83	−9.37 ± 2.06	37.49 ± 1.71
**106** (Wild)	−49.99 ± 12.33	−63.76 ± 4.65	−16.43 ± 8.70	37.68 ± 7.38
**106** (Mutant)	−50.51 ± 3.63	−66.19 ± 3.23	−8.53 ± 0.90	31.43 ± 1.38

Ensemble-averaged binding free energies (Δ*G_bind_*) were calculated using the MM-GBSA method over 1000 frames extracted from the final 100 ns of the production trajectories. Standard deviations were calculated for all energy components to assure the statistical significance of the ΔG_bind_ values. Energy components are defined as follows: Δ*E_vdw_* represents the Van der Waals interaction energy; Δ*E_elec_* represents the electrostatic interaction energy; and Δ*G_solv_* represents the polar solvation energy calculated using the VSGB 2.0 solvation model. All values are reported in kcal/mol.

## Data Availability

The data presented in this study are publicly available in the Non-Covalent_EGFR_Inhibition repository on GitHub at https://github.com/tusharpawar49/Non-Covalent_EGFR_Inhibition, accessed on 6 February 2026. This dataset includes the initial chemical library generated via the DrugEx framework, the filtered subsets of developable candidates, and the finalized set of structurally diverse compounds prioritized for structure-based evaluation. Furthermore, all raw data supporting the molecular dynamics trajectories, Free Energy Landscape (FEL) basins, and ensemble-averaged MM-GBSA binding free energy calculations for the lead candidates are hosted at the provided link to ensure the reproducibility and transparency of the structural pharmacology findings.

## Supplementary Materials

The following supporting information can be downloaded at: https://www.mdpi.com/article/10.3390/pharmaceutics18070842/s1, Figure S1: Surface Representation and Comparative Binding Modes of Lead Candidates; Figure S2: Molecular Dynamics Stability and Contact Mapping for Compound **14**; Figure S3: Molecular Dynamics Stability and Contact Mapping for Compound **88**; Figure S4: Molecular Dynamics Stability and Contact Mapping for Compound **106**; Figure S5: 2D and 3D Ramachandran Plot Analysis of EGFR-Ligand Ensembles; Table S1: Statistical Analysis of RMSD for Lead Candidates in WT and T790M EGFR; Table S2: Statistical Decomposition of Binding Free Energy via MM-GBSA and MM-PBSA; Table S3: Statistical Delineation of Free Energy Landscape (FEL) Basins; File S1: Generated_100000_Molecules.
