# Supplementary material for: Exploiting the T790M Gatekeeper: A Theoretical Blueprint for Non-Covalent Inhibition of in cis Triple-Mutant EGFR
Source: Pharmaceutics. 2026 Jul 10;18(7):842. doi: 10.3390/pharmaceutics18070842 (PMC13416115; doi:10.3390/pharmaceutics18070842)
Supplement: Supplementary file 1 [file pharmaceutics-18-00842-s001.zip › Supplementary Information.pdf]

Supplementary Information

Exploiting the T790M Gatekeeper: A Theoretical Blueprint for Non-Covalent Inhibition of in *cis* Triple-Mutant EGFR

Shrikant S. Nilewar [1], Shuvadip Khanra [1], Manav Pandya [1], Sandesh Lodha [1], Perli Kranti Kumar [2], Nagaraju Bandaru [3], Antonio Jose Naranjo-Redondo [4], Ricardo Pérez-Pastén-Borja [5,*] and Tushar Janardan Pawar [4,6,*]

[1] *Department of Pharmaceutical Chemistry, Maliba Pharmacy College, Uka Tarsadia University, Bardoli 394350, Gujarat, India;*

[2] *Department of Pharmaceutical Analysis, J.K.K. Nattraja College of Pharmacy, Kumarapalayam, 638183, Tamil Nadu, India.*

[3] *Department of Pharmacology, Sree Dattha Institute of Pharmacy Sheriguda, Ibrahimpatnam, Hyderabad, 501510, Telangana, India.*

[4] *División de Ingeniería, Universidad Anáhuac Querétaro, Circuito Universidades I, Fracción 2 S/N, Zibatá, El Marqués 76246, Querétaro, Mexico.*

[5] *Departamento de Farmacia, Escuela Nacional de Ciencias Biológicas, Instituto Politécnico Nacional, Unidad Académica Adolfo López Mateos, Del. GAM, Mexico City, 77380 Mexico.*

[6] *Centro de Investigación, Universidad Anáhuac Querétaro, Circuito Universidades I, Fracción 2 S/N, Zibatá, El Marqués 76246, Querétaro, Mexico.*

* *Correspondence: rpastenb@ipn.mx (R.P.-P.-B); tushar.pawar@anahuac.mx (T.J.P.).*

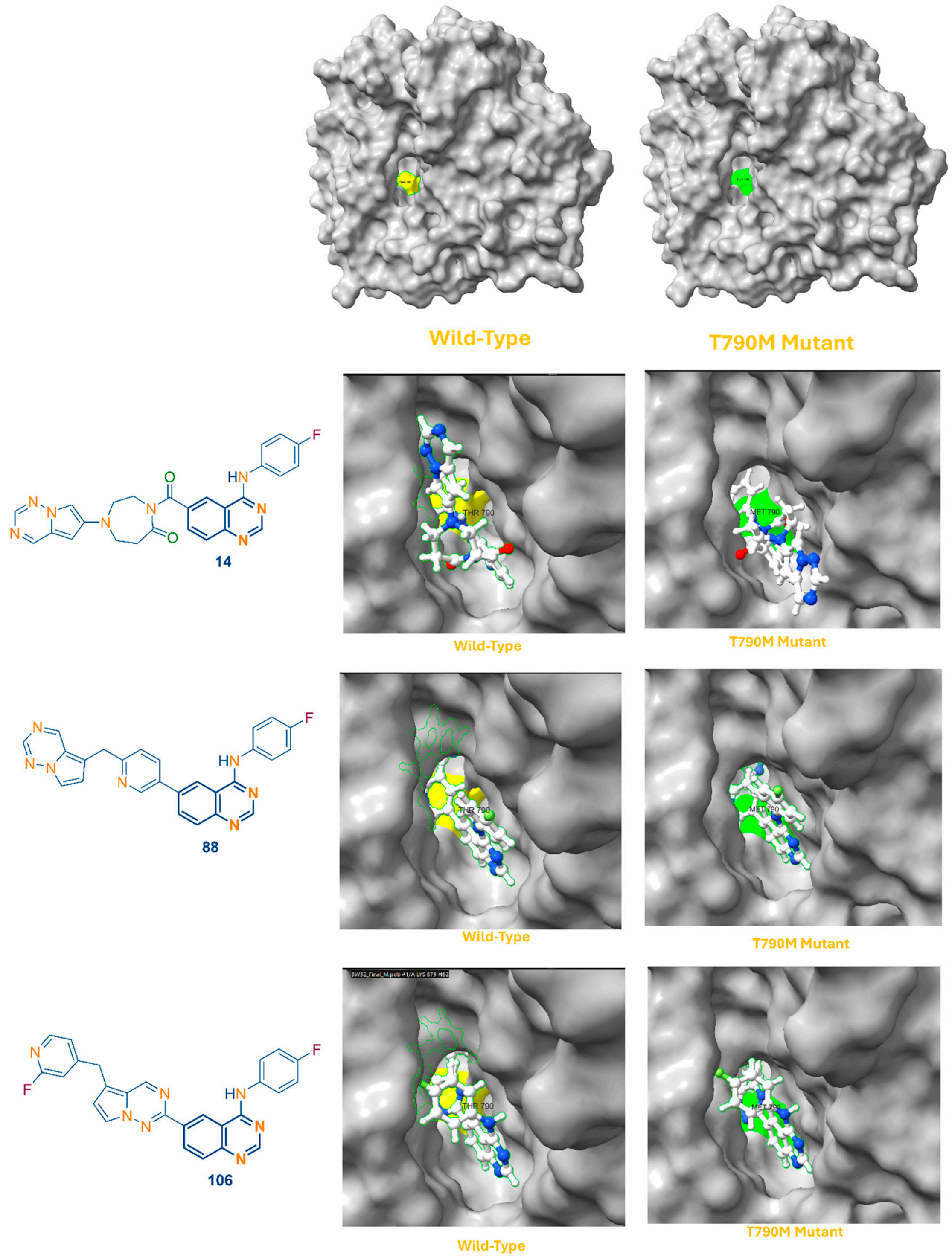

Figure S1. Surface Representation and Comparative Binding Modes of Lead Candidates.

Wild-Type-14

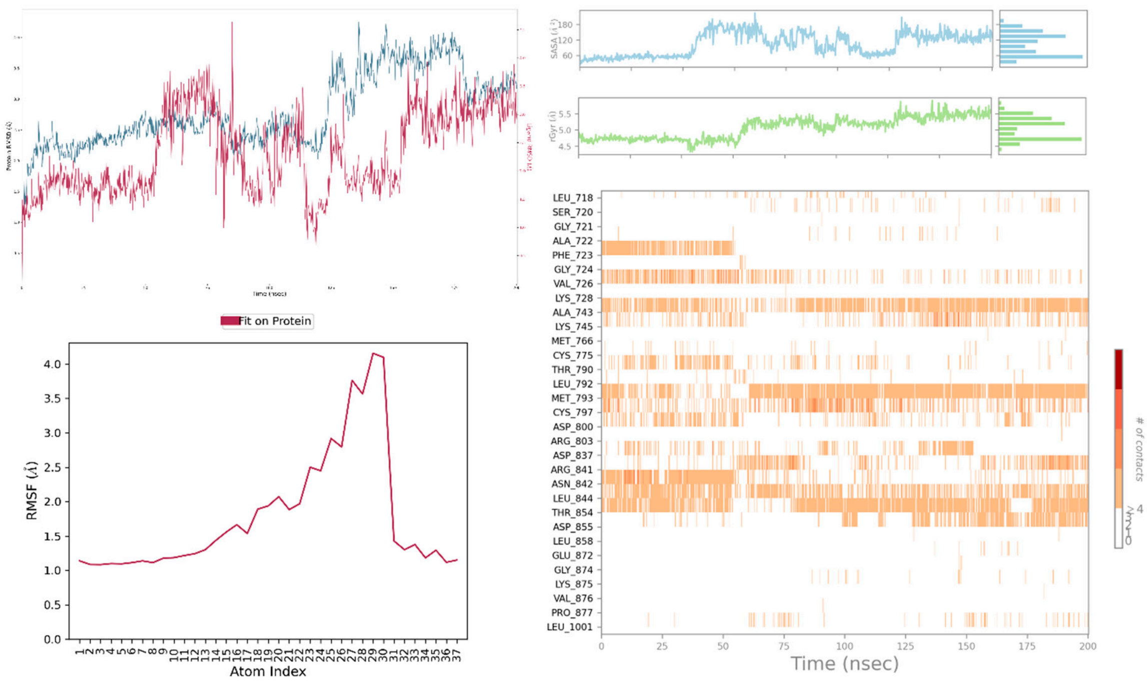

Mutated-14

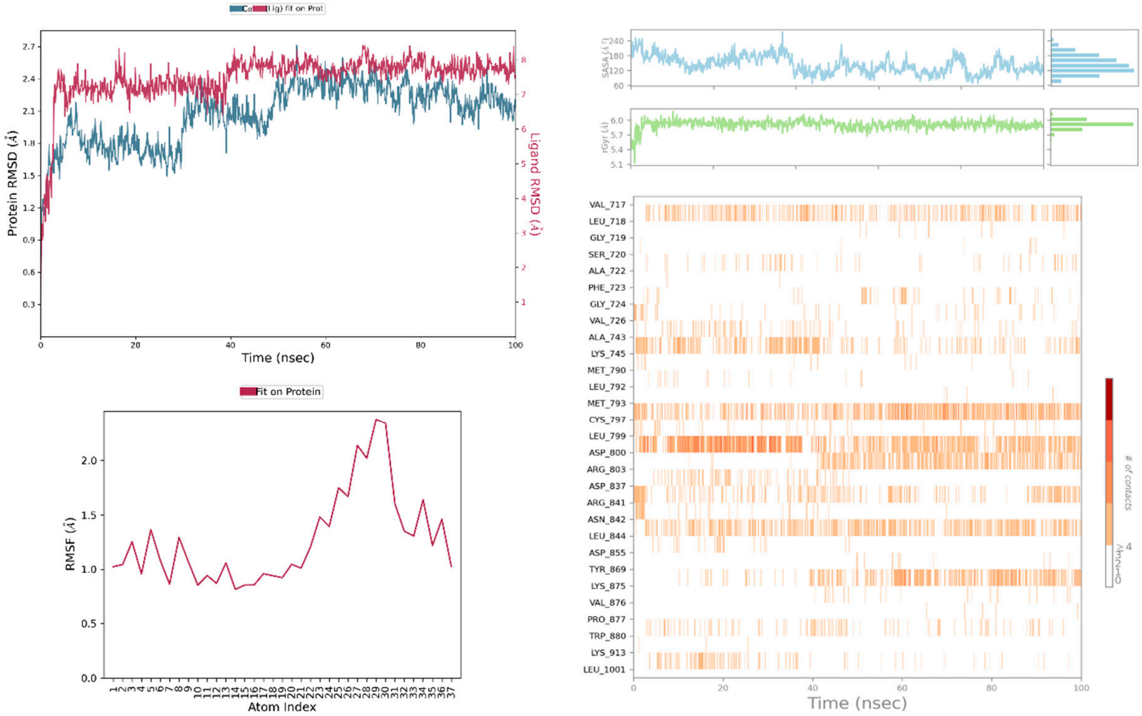

Figure S2. Molecular Dynamics Stability and Contact Mapping for Compound **14**.

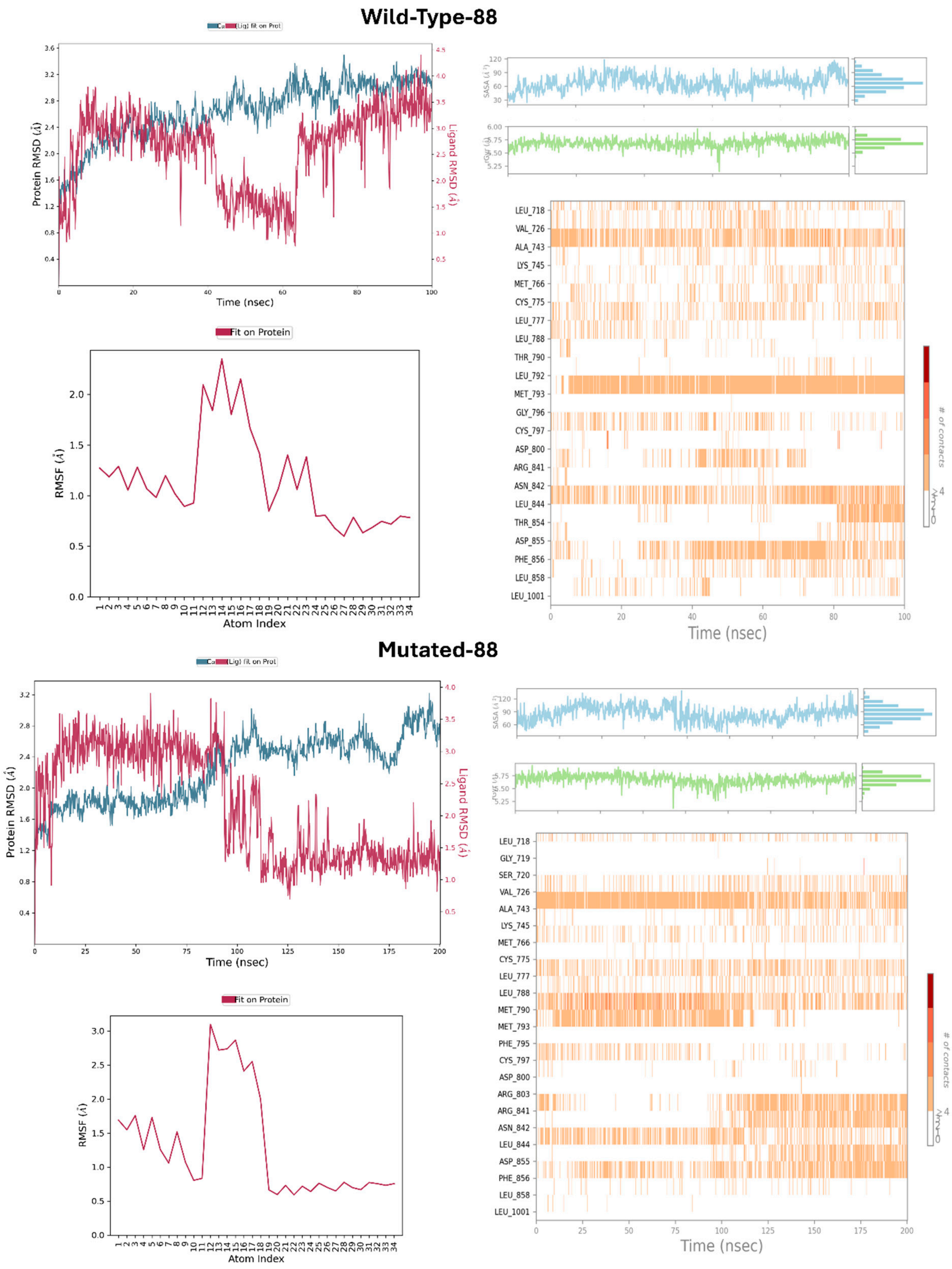

Figure S3. Molecular Dynamics Stability and Contact Mapping for Compound **88**.

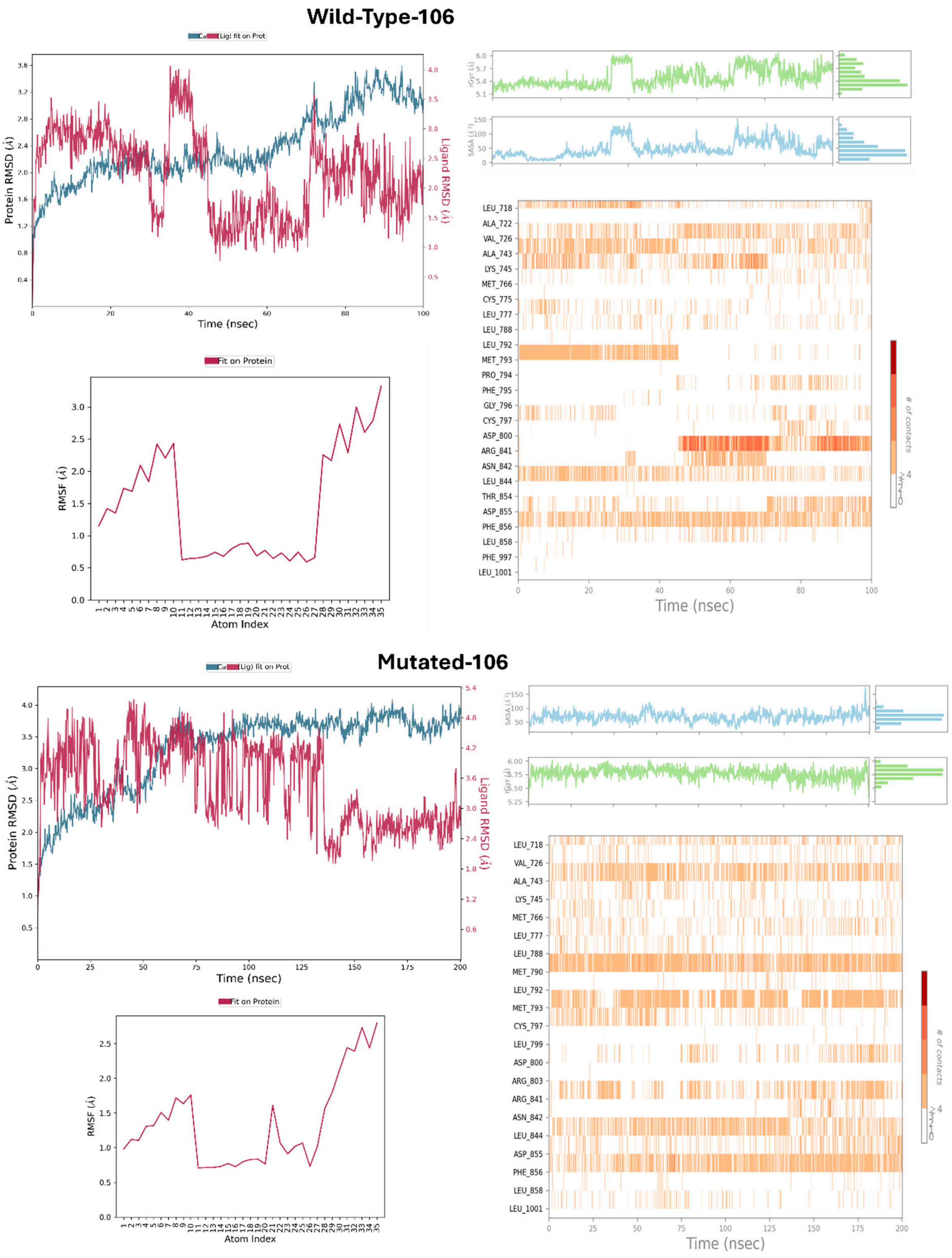

Figure S4. Molecular Dynamics Stability and Contact Mapping for Compound **106**.

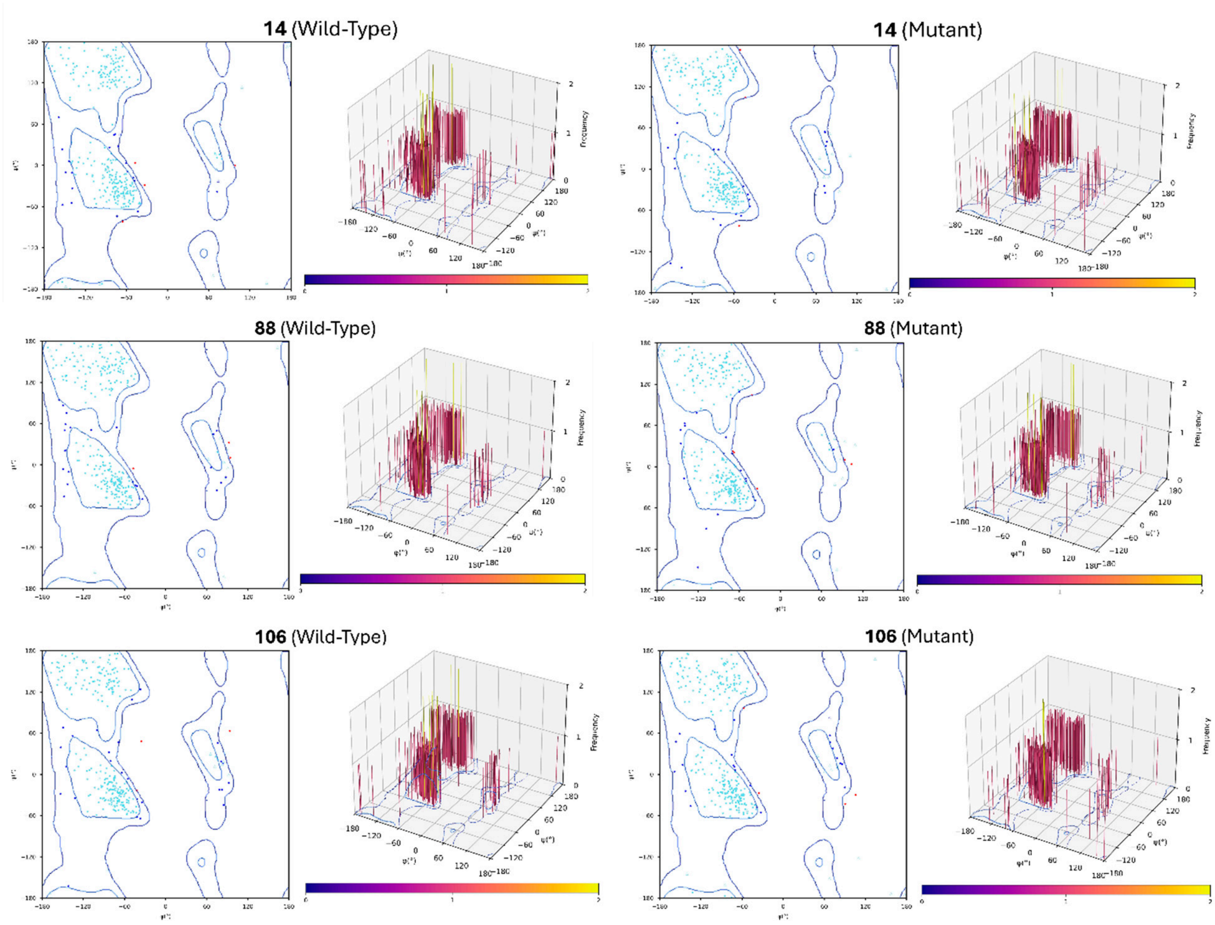

Figure S5. 2D and 3D Ramachandran Plot Analysis of EGFR-Ligand Ensembles.

Table S1. Statistical Analysis of RMSD for Lead Candidates in WT and T790M EGFR.

Compound	WT_Mean	WT_STDEV	WT_SEM	WT_Slope	MT_Mean	MT_STDEV	MT_SEM	Δ_Mean	MT_slope	P Value
**14**	2.481517928	0.698881272	0.031192598	0.031692231	7.788368526	0.216117718	0.009645806	5.306850598	-0.001095086	0.00
**88**	2.648511952	0.846455349	0.037779151	0.049161383	1.412776892	0.347378793	0.015504274	-1.23573506	-0.007083279	0.00
**88'**	2.410629482	0.263775622	0.011772882	0.004643349	3.207440239	0.285093204	0.012724332	0.796810757	-0.000830795	0.00
**106**	1.864063745	0.525970127	0.023475196	0.016764462	3.067085657	0.708132496	0.0316055	1.203021912	-0.030851381	0.00

Table S2. Statistical Decomposition of Binding Free Energy via MM-GBSA and MM-PBSA.

Compound	mode	Internal mean	Internal std	Van der Waals mean	Van der Waals std	Electrostatic mean	Electrostatic std	Polar Solvation mean	Polar Solvation std	Non-Polar Solvation mean	Non-Polar Solvation std
**14_** Wild	gb	-1.7E-05	1.3E-04	-60.3686	4.416928817	-21.00715	4.647922669	43.04046667	4.970974472	-7.25246472	0.399033881
**14_** Wild	pb	-1.7E-05	1.3E-04	-60.3686	4.416928817	-21.00715	4.647922669	53.94313333	11.37570049	30.13235	1.211291598
**14_** Mutated	gb	-1.7E-05	1.2E-04	-55.2595	1.597684304	-1.3529	3.29466028	23.72071667	2.741645058	-6.75277452	0.206960933
**14_** Mutated	pb	-1.7E-05	1.2E-04	-55.2595	1.597684304	-1.3529	3.29466028	33.19436667	3.080634484	30.02928333	1.546886353
**88** Wild	gb	-3.8E-14	8.9E-05	-66.6258	3.045956765	-11.37643333	2.326506539	33.41853333	3.251084674	-7.49369016	0.297578163
**88** Wild	pb	-3.8E-14	8.9E-05	-66.6258	3.045956765	-11.37643333	2.326506539	44.85308333	3.64121477	32.71533333	1.379658354
**88** Mutated	gb	1.7E-05	9.8E-05	-65.30605	2.833292762	-9.372316667	2.055159968	37.48988333	1.712837243	-6.99723372	0.111866907
**88** Mutated	pb	1.7E-05	9.8E-05	-65.30605	2.833292762	-9.372316667	2.055159968	48.56261667	3.325117566	31.7745	1.340693637
**106** Wild	gb	-5.0E-05	5.5E-05	-63.7649333	4.653632625	-16.43245	8.699527264	37.67953333	7.380189538	-7.46912004	0.362300984
**106** Wild	pb	-5.0E-05	5.5E-05	-63.7649333	4.653632625	-16.43245	8.699527264	54.9502	7.810524971	31.76916667	1.104897144
**106** Mutant	gb	-3.3E-05	5.2E-05	-66.18725	3.226986238	-8.533233333	0.903395397	31.43128333	1.384802561	-7.2201426	0.145806299
**106** Mutant	pb	-3.3E-05	5.2E-05	-66.18725	3.226986238	-8.533233333	0.903395397	42.5375	2.392328109	31.46835	1.725095205

Table S3. Statistical Delineation of Free Energy Landscape (FEL) Basins.

Compound	RMSD_Wild (Å)	Rg_Wild (Å)	ΔG_Wild (kJ/mol)	RMSD_Mutant (Å)	Rg_Mutant (Å)	ΔG_Mutant (kJ/mol)	ΔRMSD (Å)	ΔRg (Å)	ΔΔG (kJ/mol)
**14**	2.922	19.937	8.751	1.467	19.785	7.953	-1.455	-0.152	-0.798
**88**	2.335	20.059	13.343	2.474	19.794	15.757	0.139	-0.265	2.414
**106**	2.574	20.036	14.297	1.795	20.2	4.417	-0.779	0.164	-9.88
